# Supplementary material for: Ventral hippocampal OLM cells control type 2 theta oscillations and response to predator odor
Source: Nat Commun. 2018 Sep 7;9:3638. doi: 10.1038/s41467-018-05907-w (PMC6128904; doi:10.1038/s41467-018-05907-w)
Supplement: Supplementary file 1 — Supplementary Information [file 41467_2018_5907_MOESM1_ESM.pdf]

Ventral hippocampus OLM cells control type 2 theta oscillations and response to predator odor, Mikulovic et al.

Supplementary Figures 1 - 16

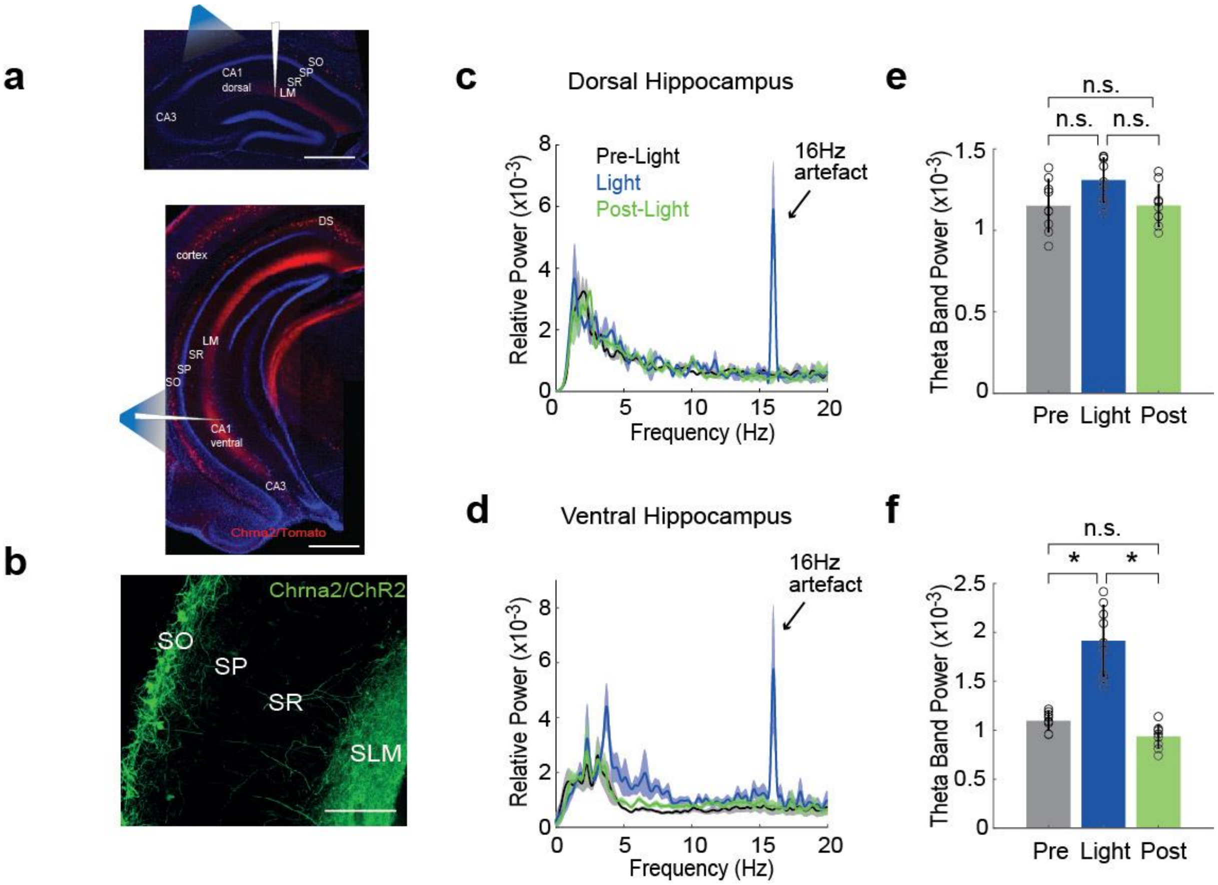

Supplementary Figure 1. OLM$^{\alpha2}$ cell stimulation at 16 Hz in vHipp, but not dHipp, induces theta band (3-8 Hz) activity in ketamine-anesthetized animals. (**a**) Recording electrode and fiber optic position in dHipp and vHipp. Scale bars: 0.4 mm (**b**) ChR2-YFP expression in CA1 of a Chrna2-Cre mouse. Scale bar: 0.1 mm. (**c,e**) Averaged power spectra showing no effect when dHipp OLM$^{\alpha2}$ cells were stimulated with 16 Hz light (n=6, n.s.= not significant). (**d,f**) Averaged power spectra showing prominent induction of theta activity (3-8 Hz) during 16 Hz OLM$^{\alpha2}$ cell stimulation in vHipp (n=9, *p<0.0001, repeated-measures ANOVA). Arrows in (**c**,**d**) point to the 16 Hz light induced artifact[1].

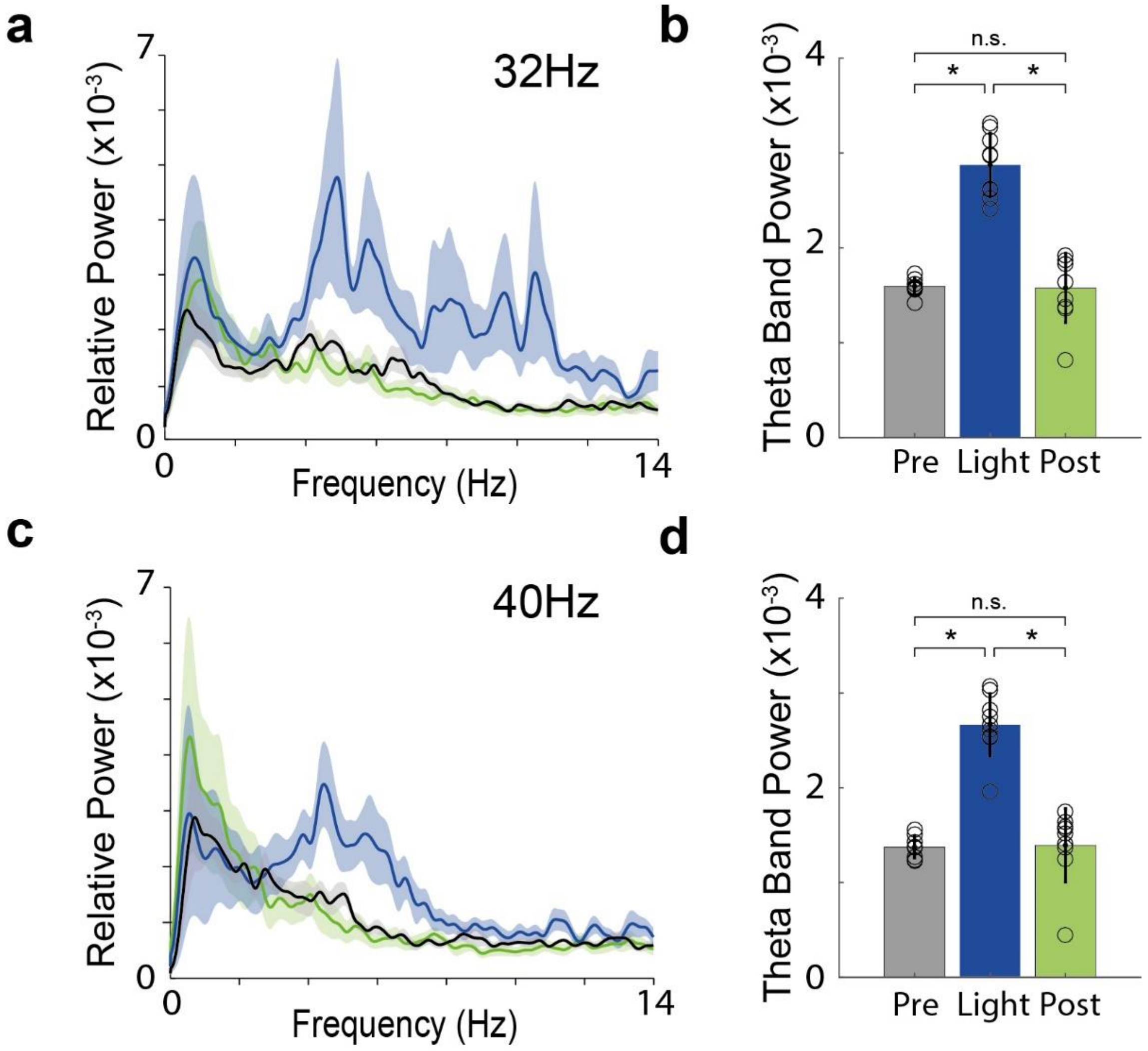

Supplementary Figure 2. Higher frequency light stimulation of OLM$^{\alpha 2}$ cells in vHipp also induces theta oscillations (3-8 Hz) in ketamine-anesthetized animals. (a) Averaged power spectra from Chrna2/ChR2 mice at 32 Hz stimulation (n=9). **(b)** Bar plots of mean theta (3-8 Hz) power. Note the significant increase in theta power during 32 Hz light stimulation (*$p<0.0001$, repeated-measures ANOVA). **(c)** Averaged power spectra from Chrna2/ChR2 mice for 40 Hz stimulation (n=9). **(d)** Bar plots of mean theta power. Note the significant increase in theta (3-8 Hz) power during 40 Hz light stimulation (*$p<0.0001$, repeated-measures ANOVA).

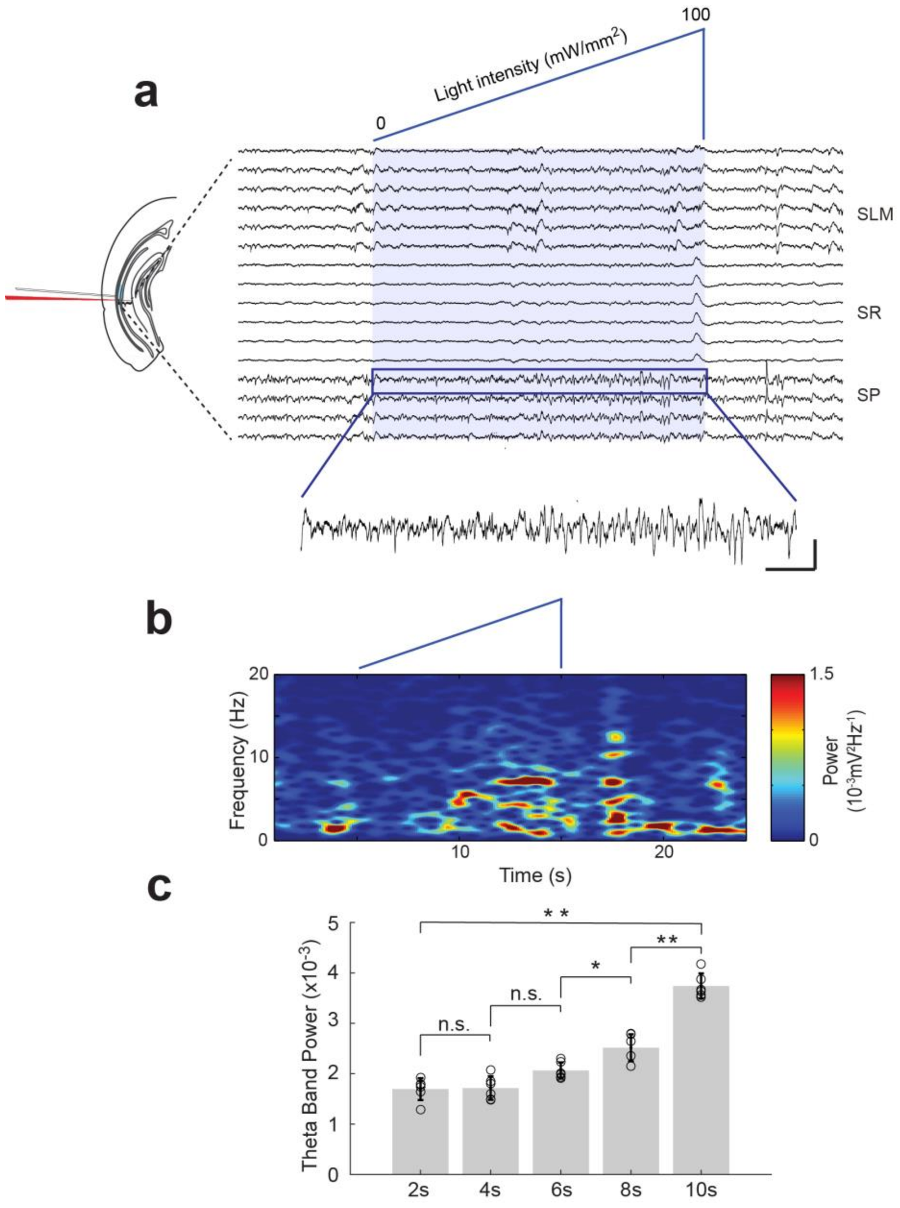

Supplementary Figure 3. Non-rhythmical stimulation of OLM$^{\alpha2}$ cells also induces theta activity (3-8 Hz) in vHipp of ketamine-anesthetized animals. (**a**) Horizontal placement of a 16-channel probe in vHipp of a Chrna2/ChR2 mouse covering the SLM, SR and SP. Traces before, during and after non-rhythmical (ramp) blue laser stimulation with light intensity from 0 to 100 mW/mm^2. The scale bar refers to the scale of the zoomed period. Scale bars: 1 s/0.05 mV. (**b**) A representative spectrogram derived from recordings upon activation of OLM$^{\alpha2}$ cells as shown in **a**. (**c**) Bar plots depicting mean theta power during light stimulation (n=6, *p<0.05, **p<0.0001, repeated-measures ANOVA). Note the increase in theta power with increased light duration.

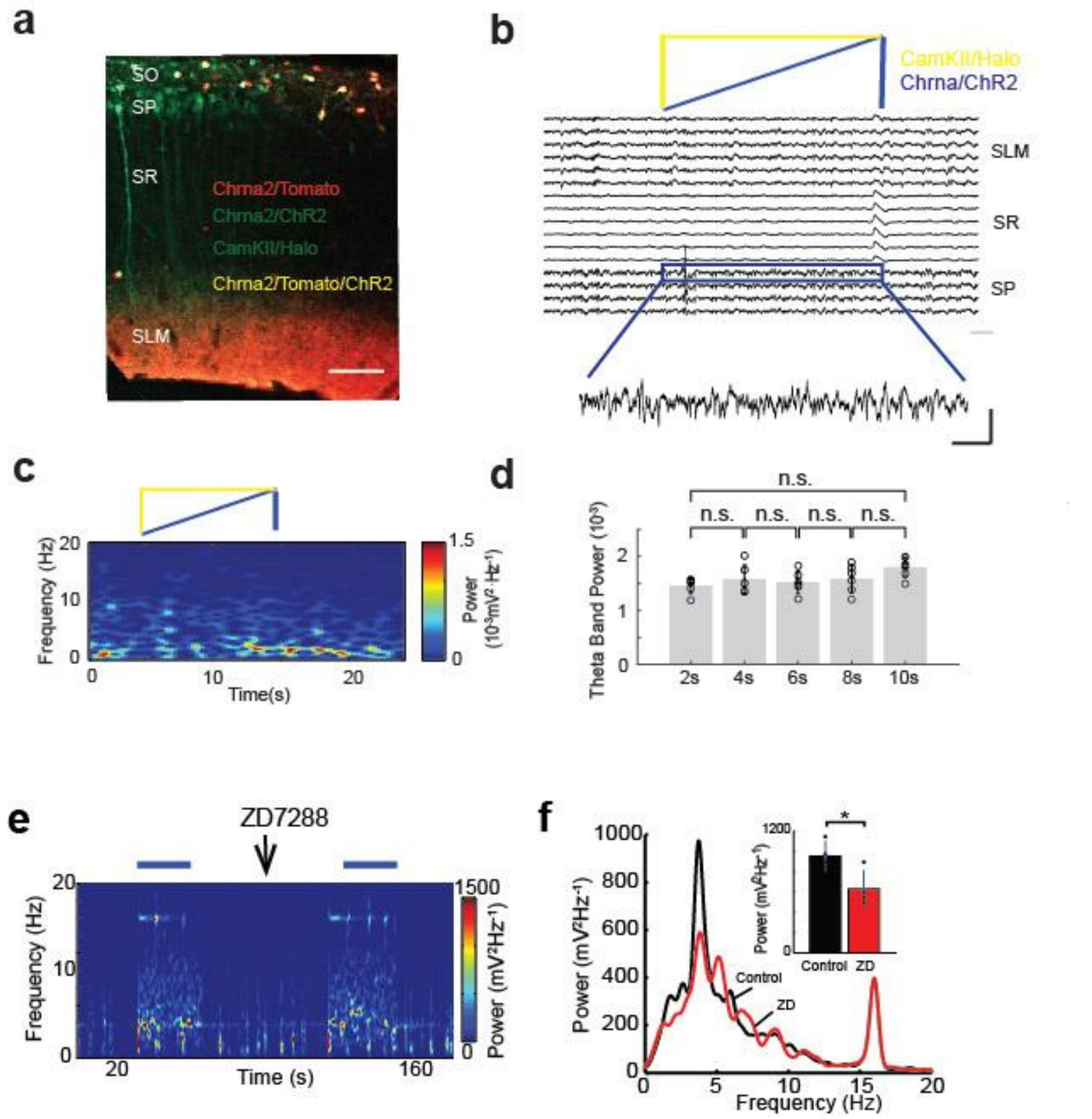

Supplementary Figure 4. OLM$^{\alpha2}$ cells require PC activity to drive theta activity (3-8 Hz) in vHipp. (**a**) Confocal microscopy image of CamKII/Halorhodopsin (reported by EGFP green fluorescence) in vHipp PCs and ChR2 in OLM$^{\alpha2}$ cells (reported by Tomato red fluorescence). Scale bar: 0.1 mm. (**b**) Simultaneous optogenetic activation of OLM$^{\alpha2}$ cells using a ramp (blue) and tonic inhibition of PCs (yellow) hinders theta activity induction (compare with Supplementary Fig. 3a,b). The scale bar refers to the scale of the zoomed period. Scale bars: 0.5 s/0.1 mV. (**c**) A representative spectrogram derived from recordings upon simultaneous activation of OLM$^{\alpha2}$ cells and tonic inhibition of PCs. (**d**) Bar plots of mean theta power showing no significant change during light stimulation (n=6, n.s. = not significant, repeated-measures ANOVA). (**e**) Spectrogram showing the effect of ZD7288 (*Ih* blocker) injection at the SLM on the power of OLM$^{\alpha2}$ cell-induced theta (16Hz sinusoid light stimulation). (**f**) Example of a power spectrum density plot showing the decrease in light-induced theta power after ZD7288 infusion. *Inset*, mean light-induced theta power before and after ZD7288 infusion (*p=0.03, n=4, t-test).

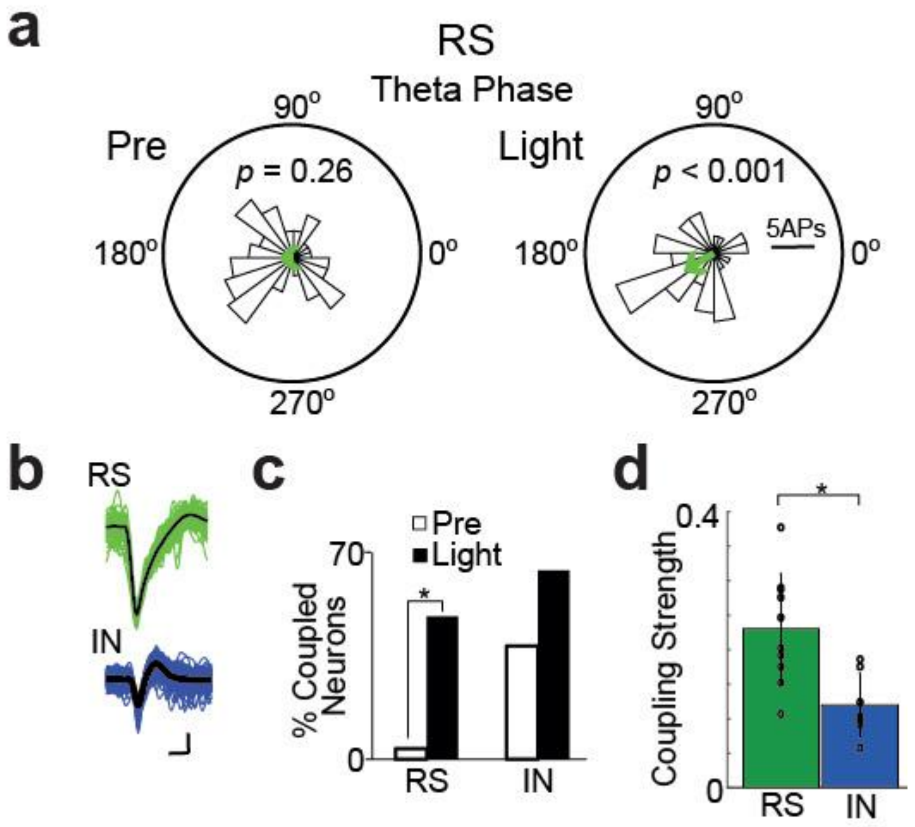

Supplementary Figure 5. OLM$^{\alpha2}$ cell stimulation couples regular spiking cell (RS) firing to theta phase. (**a**) Spike distribution over theta (3-8 Hz) phases for an RS cell before and during blue light stimulation. Scale bars: 0.5 ms/0.05 mV. (**b**) Identified waveforms for RS and interneurons (IN). (**c**), proportion of RS (n=26 from 11 mice) and IN (n=11 from 11 mice) modulated by theta before and during light (middle, *p<0.05, χ^2 test). (**d**) Mean spike-field coupling strength (|R|) during light stimulation for significantly modulated cells (n=10 and n=7 cells for RC and IN, respectively; *p=0.005, t-test).

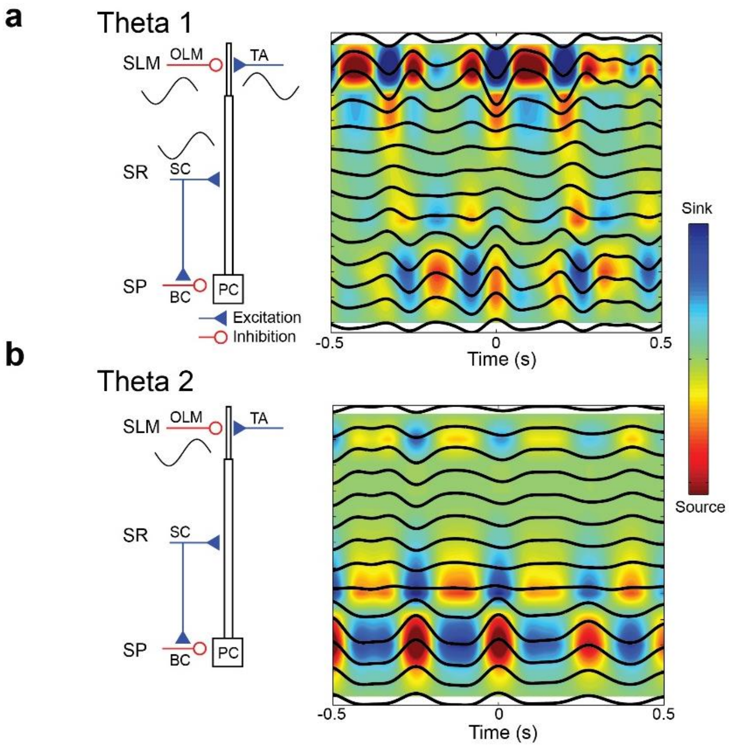

Supplementary Figure 6. The extracellular potential produced by a modeled pyramidal cell (PC) receiving theta2-like synaptic inputs attenuates the stratus lacunosum-moleculare (SLM) source. (**a**) Current source density (CSD) of extracellular potentials produced by a PC model during theta1. The PC receives in-phase rhythmical inhibition of OLM cells and Schaffer collaterals (SC) and 180° out of phase rhythmical excitation of temporammonic input (TA)[2]. SC also innervates a basket cell (BC) that inhibits the PC soma. (**b**) CSD analysis of extracellular potentials produced by a PC model during theta2. In this case, the only rhythmical input comes from OLM cells, PC receives in-phase rhythmical inhibition of OLM cells. SC and TA inputs produce tonic excitation. SR, stratum radiatum; SP, stratum pyramidale. See Online Methods for details.

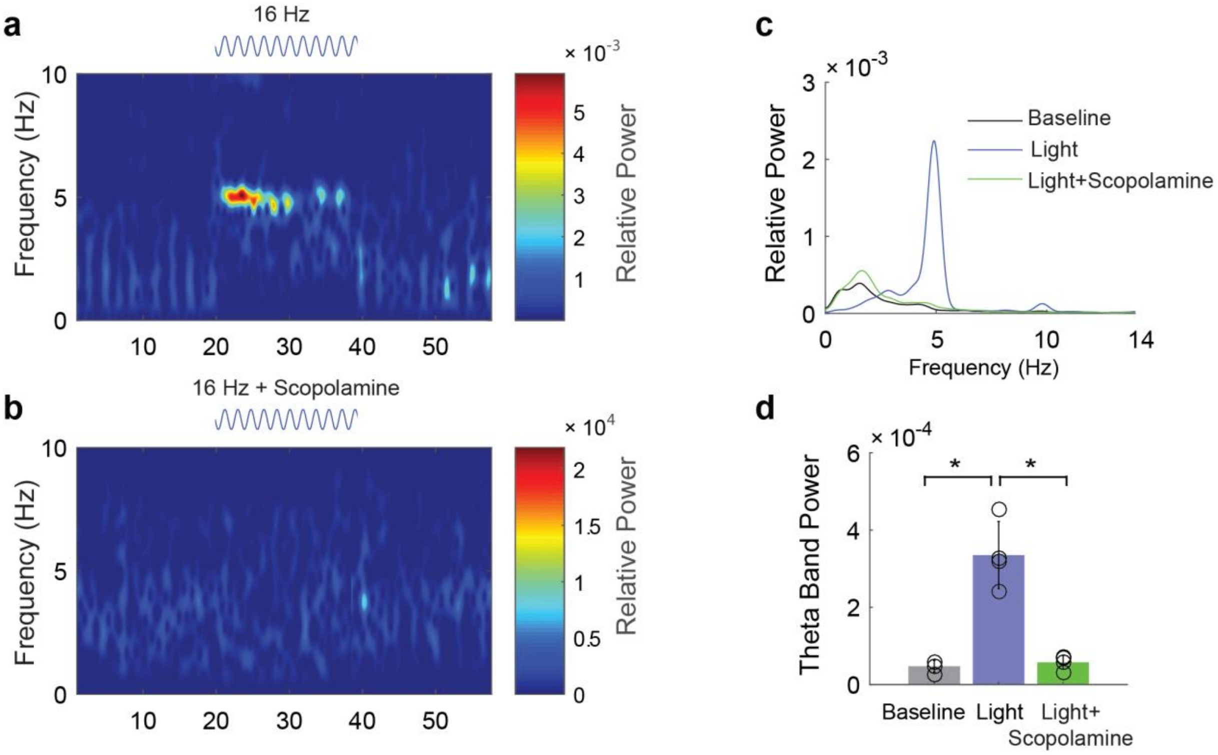

Supplementary Figure 7. Systemic scopolamine prevents theta2 oscillations induced by OLM$^{\alpha2}$ cell stimulation. (**a**) Spectrogram showing that 16 Hz light activation of OLM$^{\alpha2}$ cell induced theta activity in urethane-anesthetized animals. (**b**) Systemic scopolamine inhibits theta2 induction. (**c**) Power spectra for the recordings shown in **a** and **b**. (**d**) Bar plots of theta band power (3-8 Hz) (n=4, $p<0.0001$, repeated-measures ANOVA).

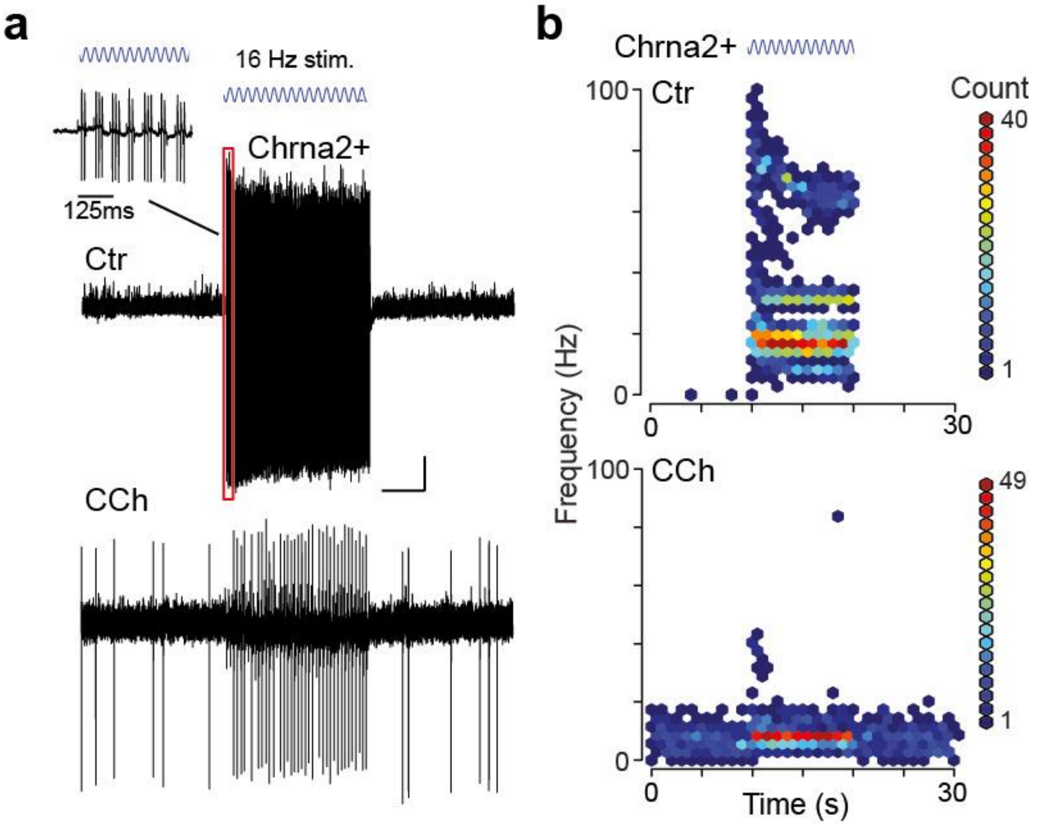

Supplementary Figure 8. *In vitro* 16 Hz light stimulation of OLM$^{\alpha 2}$ cells combined with carbachol (CCh) results in theta2-like firing. (**a**) Cell-attached recordings of an OLM cell in a slice treated with blockers of glutamatergic and GABAergic synaptic receptors (50 µM dAP5, 10 µM CNQX and 10 µM picrotoxin) showing the effects of light stimulation (16-Hz sinusoid, 10 mW/mm^2, top panel and 2 mW/mm^2 in the presence of 10 µM CCh, bottom panel). Note that at the intensity of 1 mW/mm^2 the laser pulses did not elicit spikes in the absence of CCh (data not shown). Scale bars: 0.3 s/0.5 mV. (**b**) Density plot of OLM cell instantaneous (Inst.) firing frequency as a function of time in hippocampal slices. Light stimulation (16-Hz sinusoid) time is indicated by the blue trace. The colors indicate the density of OLM cells in a specific time-frequency range. The median spike frequency during light stimulation was 8.06 Hz (interquartile range: 7.30 to 9.88 Hz; n=881 spikes, *n*=6 slices).

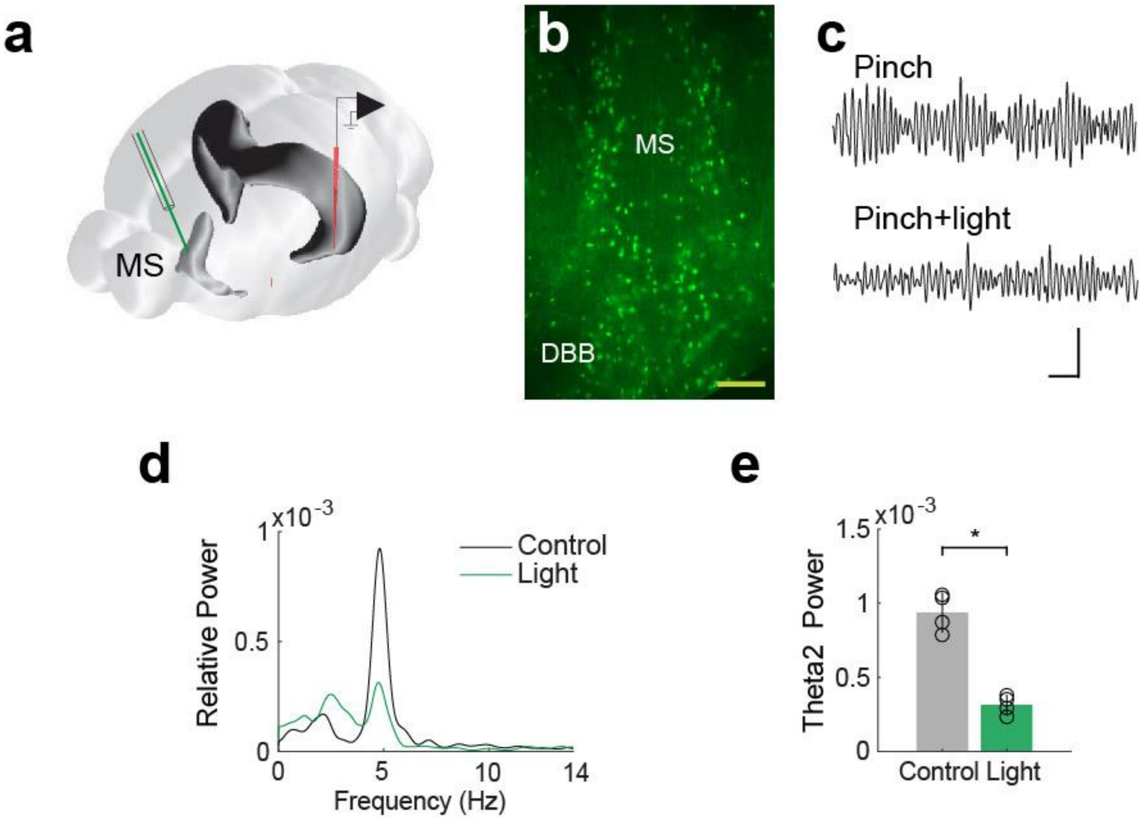

Supplementary Figure 9. Inhibition of cholinergic neurons in the medial septum/diagonal band of Broca (MS/DBB) decreases theta2 power in urethane-anesthetized animals. (**a**) Illustration of the experimental setup of MS/DBB Chat+ inhibition and vHipp LFP recording. Arch-expressing virus was injected in the MS/DBB of Chat-Cre animals. Green light was transmitted via an optic fiber in the MS/DBB, while LFP was recorded in vHipp. (**b**) Photomicrograph showing the Arch-eYFP expression in Chat-Cre MS/DBB. Scale bar: 0.2 mm. (**c-e**) Filtered traces (3-8 Hz) (Scale bars: 1 s/0.25 mV), power spectra and group statistics showing a strong decrease in power of tail pinch-induced theta2 when MS/DBB cholinergic neurons were inhibited by green light (n=4 mice, $p<0.0001$, t-test).

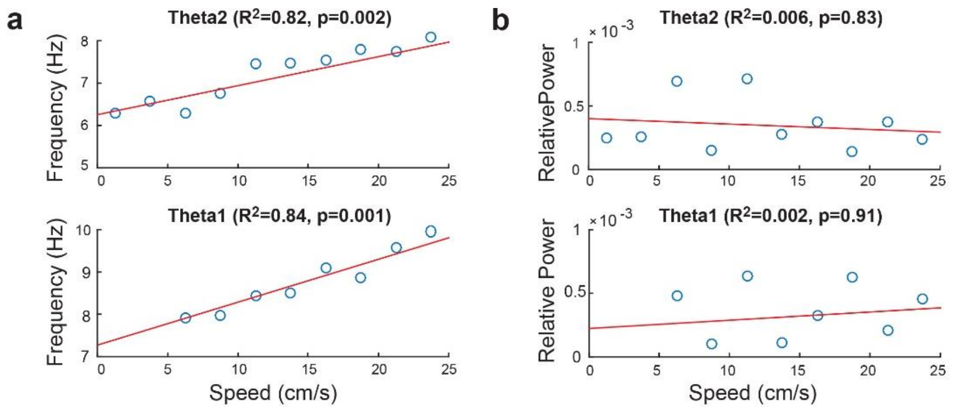

Supplementary Figure 10. Linear fitting of theta1 and induced theta2 peak frequency and amplitude as a function of animal speed. Animals (n=10) were placed on a treadmill and the speed was progressively increased from 5 to 25 cm/s in 2.5 cm/s steps. At each speed, a 60-s session during OLM$^{\alpha 2}$ cell stimulation was recorded, and the peak frequency and amplitude of both theta ranges were extracted. Each dot represents the average value for all tested animals at the analyzed speed. The theta2 peak was defined as the lower frequency peak, and the theta1 peak as the higher frequency peak (see Online Methods). Statistical line fitting (Least Square Regression) shows that (**a**) the peak frequency for both thetas was significantly correlated with the treadmill speed, while (**b**) the average peak amplitude was not.

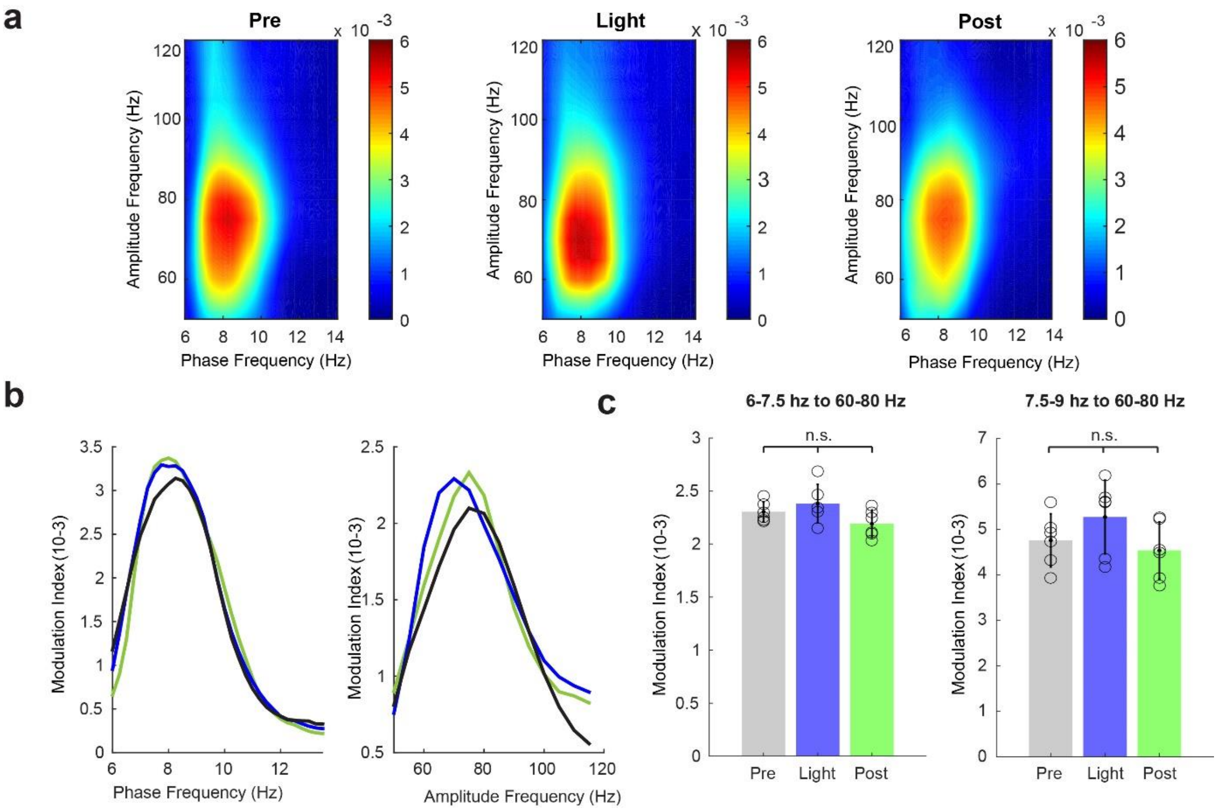

Supplementary Figure 11. OLM$^{\alpha2}$ cell stimulation does not affect theta-gamma comodulation in the dHipp. (**a**) Phase-amplitude comodulation maps for an electrode placed in dHipp SR of a Chrna2/ChR2 mouse running on a treadmill at 10 cm/s. Periods of 60 seconds before, during and after OLM$^{\alpha2}$ cell stimulation were analyzed. (**b**) Peak modulation index values for phase (theta) and amplitude (gamma) frequencies for the example in **a**. (**c**) No significant change was observed in 60-80 Hz amplitude coupling to neither 6-7.5 Hz nor 7.5-9 Hz phase (n=6, n.s. = not significant, repeated-measures ANOVA).

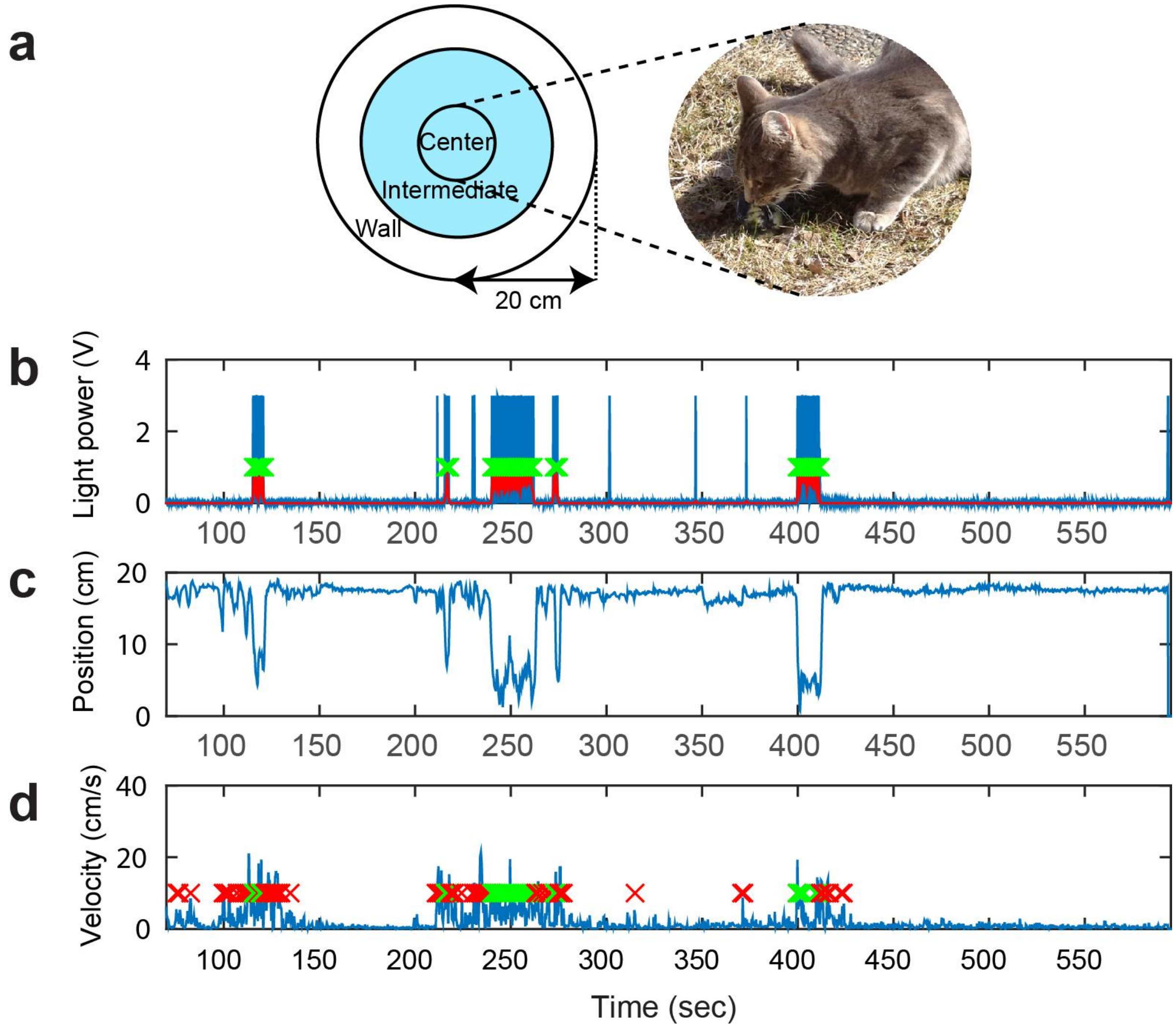

Supplementary Figure 12. **Behavior experimental setup.** (**a**) The circular arena was divided into 3 zones: Wall, Intermediate and Center. Cat hair from a verified hunter (the picture shows the actual cat used in this study - see Online Methods) was placed in the Center zone. Light was activated in the Intermediate and Center zones. (**b-d**) Laser activation timings, position (0 is the Center) and velocity were simultaneously recorded during the test. Green crosses represent detected movement during laser activation, whereas red crosses represent detected movement when the laser was off.

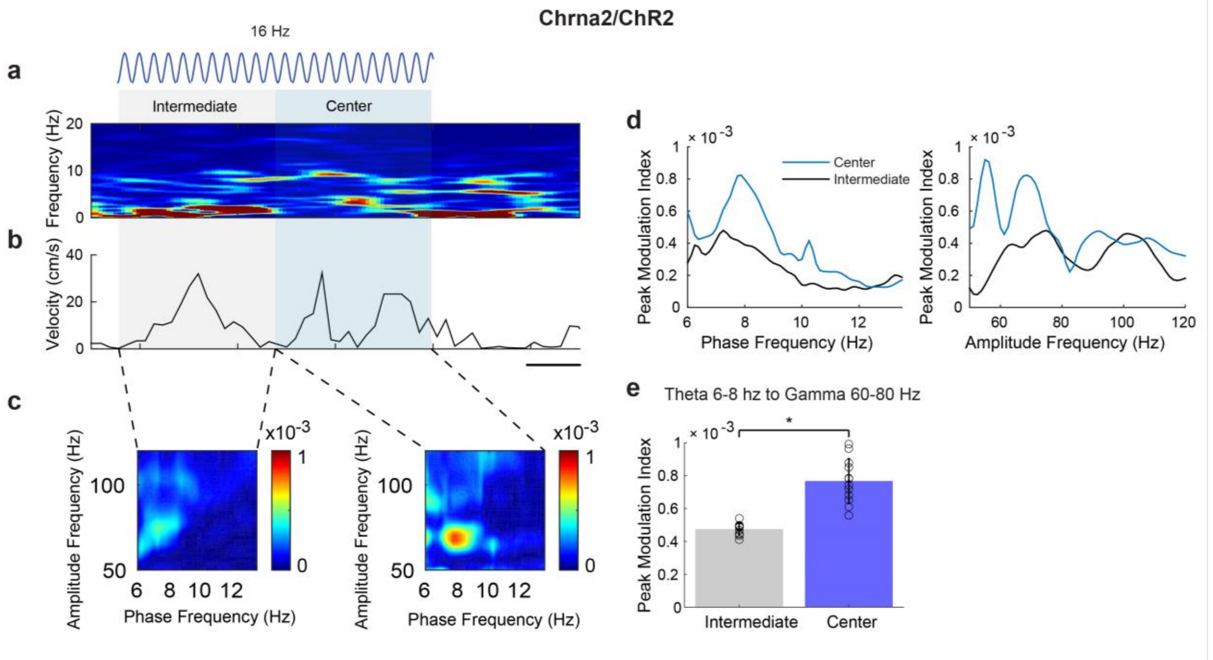

Supplementary Figure 13: Theta2–gamma comodulation increases in the vHipp of Chrna2/ChR2 animals when approaching the Center zone. (**a-c**) Spectrogram, velocity and comodulograms showing that, when animals ran with similar velocities across the Intermediate and Center zones, theta2–gamma comodulation increased towards the Center of the arena. Scale bar: 5 s. (**d**) Peak modulation indices for theta and gamma frequencies. (**e**) Group statistics (n=12, p<0.0001, t-test).

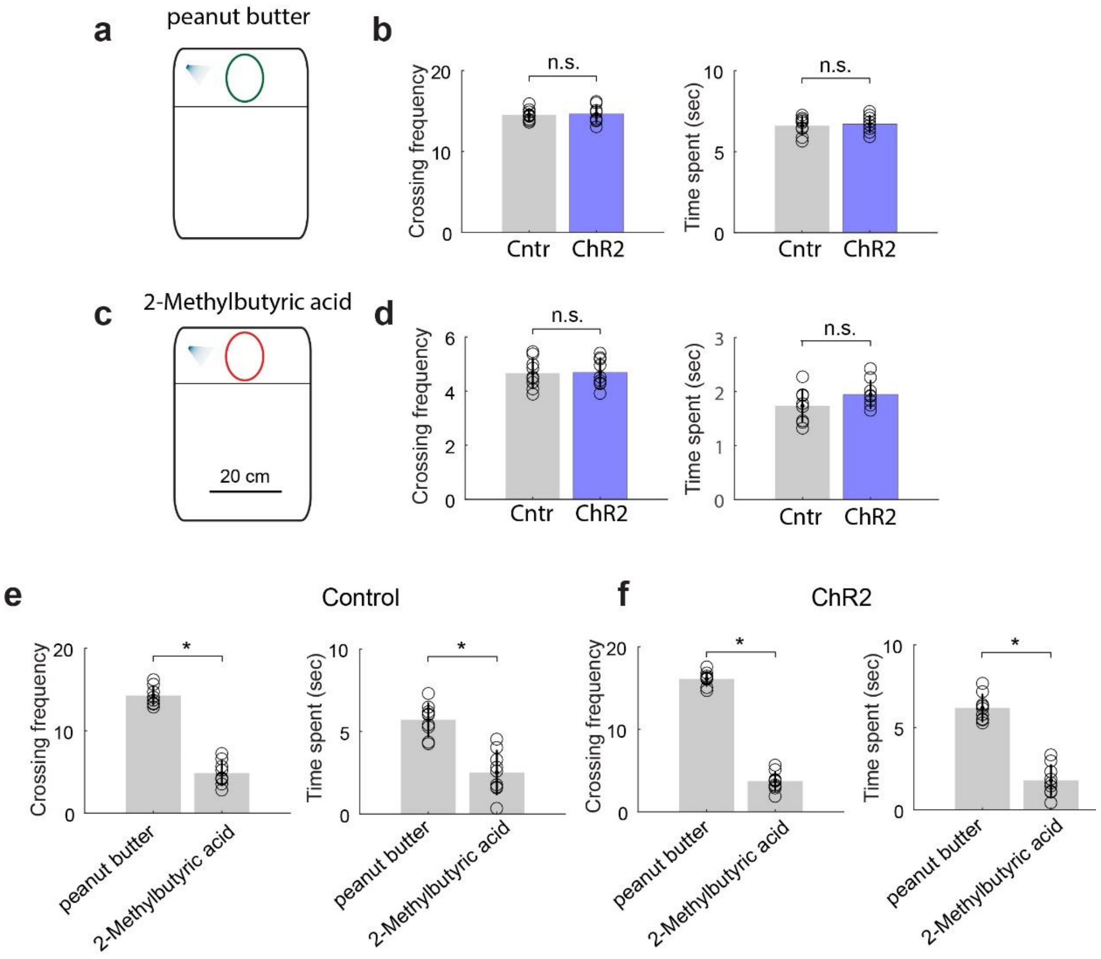

Supplementary Figure 14. Chrna/ChR2 mice display normal smell behavior. (**a-d**) No difference (n.s. = not significant, t-test) between Control (Chrna2-Cre animals injected with eYFP virus in vHipp) and Chrna2/ChR2 (injected with ChR2 virus in vHipp) animals (n=9 per group) in frequency and time spent in the zone where non-aversive (peanut butter) (**a**,**b**) and aversive scents (2-Methylbutyric acid) (**c**,**d**) were placed. Blue light (16 Hz sinusoid) was applied for both Control and Chrna2/ChR2 animals in the proximity of the smell stimuli, as depicted in **a** and **c**. (**e**,**f**) Both Control and Chrna2/ChR2 groups distinguish between the non-aversive and aversive scents (n=18 mice, $p<0.0001$, t-test).

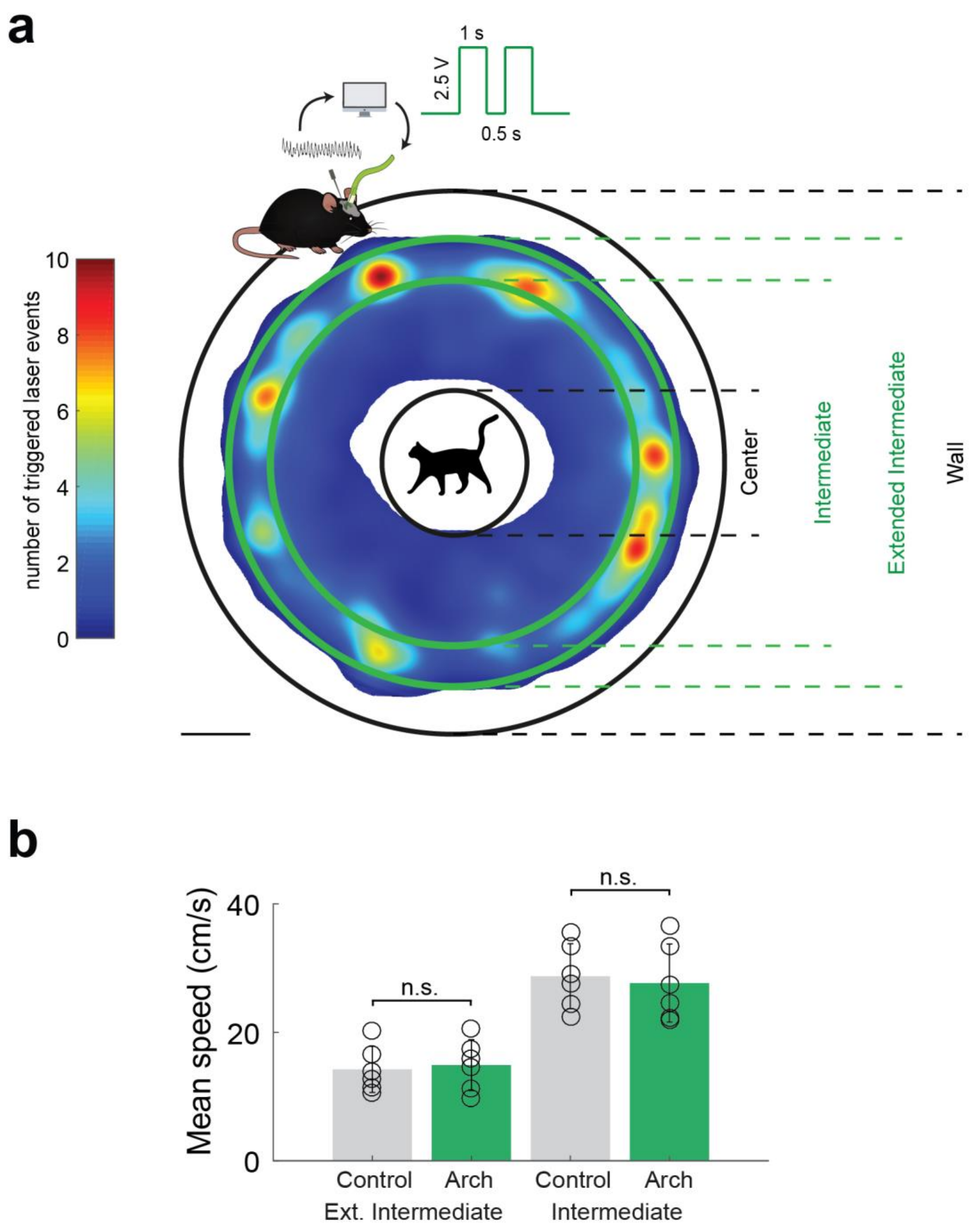

Supplementary Figure 15. Experimental setup of a closed-loop real-time inhibition of the naturally occurring theta2 in moving animals in the predator odor test. (**a**) Upon theta2 (6-8 Hz) and animal movement (velocity > 5 cm/s) detection (see Online Methods), two light pulses (1-s duration, 500-ms inter-pulse interval) were generated to inhibit OLM$^{\alpha2}$ cells in Chrna2-Cre/Arch animals. The same method was also used in Control (Chrna-Cre animals injected with eYFP control virus, see Fig. 7) and immobile animals (see Supplementary Fig. 19). The heatmap shows the density of triggered laser events in a Chrna2-Cre/Arch animal for the duration (10 minutes) of a predator odor test. Upon the post-experiment analysis, we observed that triggered events occurred predominantly in an area that extends ~ 4 cm from the border of the Intermediate zone. For further analysis of risk-tasking behavior, we thus defined the zone as Extended Intermediate. Scale bar: 5 cm. The cat and mouse cartoon was produced by the authors using the free computer programs GIMP and

Inkscape. (**b**) Bar plots showing average speed in Control and Chrna2-Cre/Arch animals in the Extended Intermediate and Intermediate zones (n=6, n.s. = not significant, t-test).

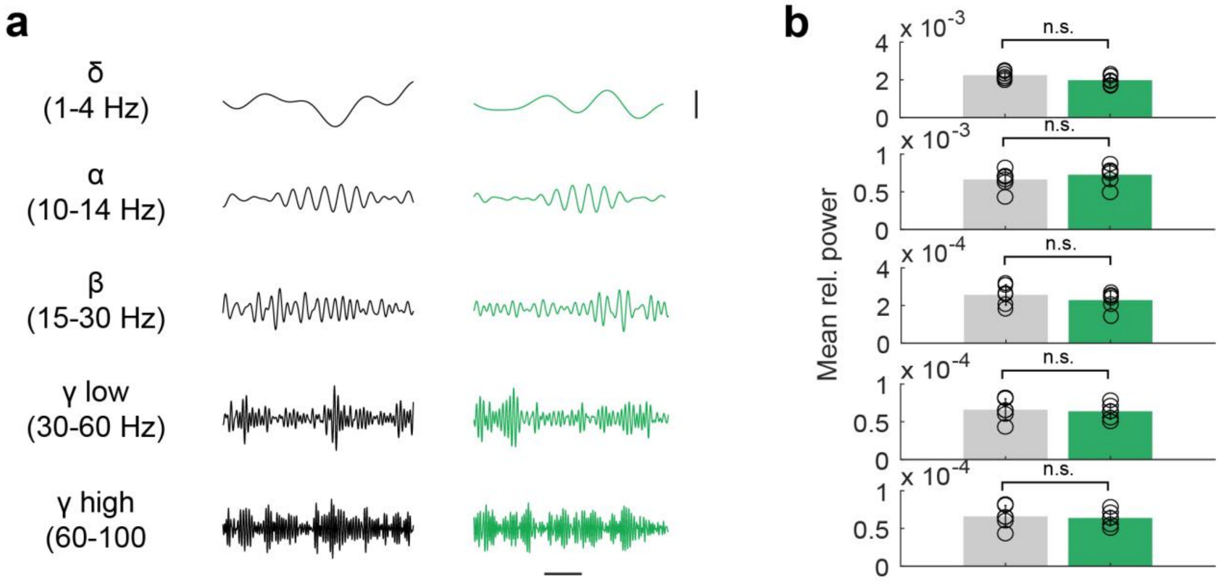

Supplementary Figure 16. OLM$^{\alpha 2}$ cell silencing specifically reduces power of theta2. (**a,b**) OLM$^{\alpha 2}$ cell silencing did not affect other oscillation frequencies[3]: δ (1-4 Hz), α (10-14 Hz), β (15-30 Hz), γlow (30-60 Hz), γhigh (60-100 Hz) (n=6, n.s. = not significant, t-test, Scale bars: 0.2 s/0.1 mV).

References

1. Mikulovic, S. *et al.* On the photovoltaic effect in local field potential recordings. *Neurophotonics* **3,** 015002 (2016).
2. Brankack, J., Stewart, M. & Fox, S. E. Current source density analysis of the hippocampal theta rhythm: associated sustained potentials and candidate synaptic generators. *Brain Res.* **615,** 310–327 (1993).
3. Boyce, R., Glasgow, S. D., Williams, S. & Adamantidis, A. Causal evidence for the role of REM sleep theta rhythm in contextual memory consolidation. *Science* **352,** 812–816 (2016).
4. Chittajallu, R. *et al.* Dual origins of functionally distinct O-LM interneurons revealed by differential 5-HT(3A)R expression. *Nat. Neurosci.* **16,** 1598–1607 (2013).
5. Sekulić, V. & Skinner, F. K. Computational models of O-LM cells are recruited by low or high theta frequency inputs depending on h-channel distributions. *eLife* **6,** e22962 (2017).
6. Fuhrmann, F. *et al.* Locomotion, theta oscillations, and the speed-correlated firing of hippocampal neurons are controlled by a medial septal glutamatergic circuit. *Neuron* **86,** 1253–1264 (2015).
7. Ahmed, O. J., Mehta, M.R. Running speed alters the frequency of hippocampal gamma oscillations. *J Neurosci.* 32(21):7373-83 (2013).
