# Supplementary material for: Ventral hippocampal OLM cells control type 2 theta oscillations and response to predator odor
Source: Nat Commun. 2018 Sep 7;9:3638. doi: 10.1038/s41467-018-05907-w (PMC6128904; doi:10.1038/s41467-018-05907-w)
Supplement: Supplementary file 3 — Description of Additional Supplementary Files [file 41467_2018_5907_MOESM3_ESM.pdf]

Description of Additional Supplementary Files:

Supplementary Movie 1: 3D CLARITY imaging showing a gradient-like distribution of $OLM^{\alpha2}$ cells (red) along the dorsoventral hippocampal axis. This movie also shows that $OLM^{\alpha2}$ cells are a subpopulation of Somatostatin (green) positive cells.
